# The analysis of factors influencing patient choice of healthcare providers between tertiary hospitals and community clinics

**DOI:** 10.3389/fpubh.2025.1510311

**Published:** 2025-02-04

**Authors:** Silin Wu, Zhaoxia Lei, Tinglian Liu, Lan Chen, Yang Qin

**Affiliations:** ^1^Clinical School of North Sichuan Medical College, Nanchong, China; ^2^Department of General Practice, The General Hospital of Western Theater Command, Chengdu, Sichuan, China; ^3^Longtan Community Health Service Centre, Chengdu, Sichuan, China; ^4^Shaheyuan Community Health Service Centre, Chengdu, Sichuan, China

**Keywords:** medical selection behavior, hierarchical medical system, family doctor system, having first consultations in primary care, influencing factors

## Abstract

**Background:**

The uneven distribution of medical resources in China has led to persistently low utilization rates of primary healthcare institutions. The tiered healthcare delivery system aims to optimize resource allocation and strengthen primary care, yet patient preferences for tertiary hospitals hinder its effective implementation. Understanding the factors influencing healthcare-seeking behaviors is crucial for improving policies and promoting system efficiency.

**Objective:**

To analyse the factors influencing patient choices between a tertiary hospital and a community health service center in Chengdu and provide recommendations for advancing the tiered healthcare system.

**Methods:**

A random sampling method was used in August 2023 to survey patients at a tertiary hospital (Group A) and a community health service center (Group B). The survey assessed demographics, health status, factors influencing provider choice, and awareness of the family doctor system. Chi-square, t-tests, or Wilcoxon rank-sum tests were used for group comparisons, while logistic regression identified factors associated with primary care visits.

**Results:**

Among 865 valid responses (Group A: 420; Group B: 445; 92.02% response rate), Group A had significantly higher education levels and household incomes (*p* < 0.001), while Group B had higher chronic disease prevalence and family doctor contract rates (71.5% vs. 59.3, 44.5% vs. 25.5%; both *p* < 0.01). Positive factors for choosing community healthcare included better equipment and medication availability (46.9%), lower costs with higher reimbursement (45.0%), and convenient transport (41.2%). Negative factors included distrust in community care quality (39.1%) and limited familiarity with family doctors (32.8%). Logistic regression indicated that being over 60 years old (OR: 1.94, CI: 1.02–3.69) and awareness of the tiered healthcare system (OR: 2.48, CI: 1.56–3.96) were significant factors for seeking primary care.

**Conclusion:**

Patients with higher education and income prefer tertiary hospitals, while chronic disease patients are more likely to utilize community care. Low family doctor contract rates and trust in community healthcare quality remain barriers. Strengthening community resources and promoting the tiered healthcare system could improve patient participation and alleviate pressure on tertiary hospitals.

## 1 Introduction

Reducing healthcare costs, optimizing medical resource utilization, and improving accessibility and equity in healthcare are persistent challenges for healthcare reforms worldwide (1–5). In China, the significant demand for healthcare services contrasts sharply with the uneven distribution of medical resources (6, 7). To address these issues, the government launched a nationwide healthcare reform in 2009, introducing the tiered healthcare delivery system as a key initiative to optimize resource allocation and enhance primary care accessibility (8).

The tiered healthcare delivery system is designed to guide patients to select healthcare institutions based on their conditions, promoting primary care at grassroots institutions and seamless referrals across levels (9–11). This system classifies healthcare institutions into tertiary hospitals, secondary hospitals, and community health service centers, each with distinct roles. Tertiary hospitals manage complex and severe conditions, secondary hospitals handle recovery and stable severe cases, and community health service centers focus on prevention, health management, and common illnesses (12). Despite these measures, many patients still prefer tertiary hospitals, leading to resource overutilization and persistently low utilization rates of primary care institutions (13).

To encourage the use of primary care, the family doctor system was introduced, aiming to provide continuous, comprehensive, and convenient services through contracts between family doctors and residents (14–17). Chengdu, as a pilot city for the tiered healthcare system, has implemented initiatives such as hospital reforms, bed capacity control, private sector involvement, universal health insurance, and integration mechanisms. By 2024, Chengdu had established 146 medical consortia, urban medical groups, and a bidirectional referral platform while prioritizing outpatient appointments and inpatient beds for primary care (18–20).

Despite these advancements, disparities remain in resource allocation and service quality between tertiary hospitals and community health centers (21). Community health service centers are vital for managing common illnesses and older people health but face challenges such as low family doctor contracting rates, limited trust in family doctors, and variability in service capacity (22–24).

Previous research highlights the complexity of factors influencing patients’ healthcare-seeking behaviors, including geography, service quality, trust, economic burden, and health status (13). However, most studies focus on single-tier institutions, lacking comparisons across healthcare levels. Additionally, there is limited exploration of how the family doctor system affects patient behavior, particularly among different demographic groups.

This study addresses these gaps by analyzing healthcare-seeking behaviors and influencing factors at a tertiary hospital and a community health service center in Chengdu. It also examines patients’ awareness of the family doctor system and its role in shaping healthcare choices.

## 2 Methods

### 2.1 Study design

This cross-sectional study was conducted in Chengdu, Southwest China, in August 2023. A random sampling method was employed to survey two patient groups: Group A (attending a tertiary general hospital) and Group B (attending a community health service center). Inclusion criteria: (1) Adults (≥18 years old) who voluntarily participated in the study and were able to clearly express their thoughts. (2) Outpatients attending general practice or internal medicine-related departments. Exclusion criteria: patients with unstable conditions or those unable to independently complete the questionnaire.

### 2.2 Questionnaire

A structured questionnaire was developed by the research team, comprising three sections: (1) Sociodemographic Information: Including age, gender, education level, and monthly income. (2) Health Status and Healthcare-Seeking Habits: Questions on health status (e.g., presence of chronic diseases) and healthcare-seeking behaviors (e.g., choice of primary consultation institutions). (3) Awareness and Contracting Status of the Family Doctor System: This section assessed respondents’ knowledge and experiences with the family doctor system across four dimensions: service experience, quality evaluation, service efficiency and accessibility, and referral-related issues, rated using a 5-point Likert scale.

To ensure the questionnaire’s validity, two rounds of expert reviews (with family doctor teams and tertiary hospital specialists) were conducted, followed by a pilot survey involving 10 randomly selected patients. Reliability testing in SPSS 26.0 yielded a Cronbach’s *α* of 0.887, indicating good internal consistency. Based on feedback, minor adjustments were made to improve clarity and relevance.

### 2.3 Data collection

This study received ethical approval from the Ethics Committee of Jinjian People’s Hospital and Shaheyuan Community Health Service Center, Chengdu (approval number: 2023EC2-3). Patients provided written informed consent and were briefed about the study. Data were collected using both the “Wenjuanxing”-APP online survey platform and paper-based questionnaires. Surveys were conducted in the presence of research staff, who clarified any queries raised by respondents. For participants with reading difficulties, assistance was provided without influencing their responses. A total of 940 questionnaires were distributed, yielding 865 valid responses (Group A: 420; Group B: 445), with an effective response rate of 92.02%.

### 2.4 Quality control

The survey team consisted of postgraduate medical students and healthcare professionals from the community health service center. All team members underwent standardized training before the survey to ensure consistency. Surveyors did not interfere with questionnaire completion but provided clarification for any questions raised by respondents. For older people patients or those with reading difficulties, surveyors assisted in completing the questionnaire. After data collection, all responses were subjected to double-checking to ensure accuracy and to eliminate invalid responses.

### 2.5 Statistical analysis

Data verification, cleaning, and organization were performed using Excel, and invalid questionnaires were excluded. Statistical analysis was conducted using SPSS 26.0 software. (1) Categorical data were summarized as frequencies (%) and analyzed with the *χ*^2^ test. (2) Continuous data were expressed as mean ± standard deviation (x ± s) and compared using independent samples *t*-tests. (3) Ordinal data were analyzed using the Wilcoxon rank-sum test. (4) Binary logistic regression was applied to identify factors influencing the likelihood of patients in tertiary hospitals seeking primary care as their first contact. Statistical significance was set at *p* < 0.05.

## 3 Results

### 3.1 Comparison of basic characteristics between the two groups

Among the 420 patients attending the tertiary general hospital, 308 (73.3%) were from urban areas, 62 (14.8%) were from rural areas, and 50 (11.9%) were from suburban regions. In contrast, among the 445 patients attending the community health service center, 419 (94.16%) were residents of the community, while 26 (5.84%) were from other regions. The results show no significant differences between the two groups in terms of gender and age (*p* > 0.05). However, significant differences were observed in education level, average monthly household income, chronic disease prevalence, and family doctor contracting rates (*p* < 0.001) (Table 1).

**Table 1:** Comparison of basic characteristics between tertiary hospital patients and community health center patients [*n* (%)].

Variables	Tertiary hospital patients (*n* = 420)	Community health center patients (*n* = 445)	Statistic value	*p*-value
Gender			0.96^a^	0.34
Male	171 (41.4%)	167 (37.5%)		
Female	249 (58.6%)	278 (62.5%)		
Age (Mean ± SD)	54.50 ± 13.59	53.63 ± 13.58	−0.842^b^	0.4
Education level			−8.27^c^	<0.001
Primary school or below	19 (4.5%)	100 (22.5%)		
Middle school	52 (12.5%)	94 (21.1%)		
High school (including technical school)	106 (25.2%)	59 (13.3%)		
Bachelor’s degree/college or above	243 (57.9%)	192 (43.1%)		
Monthly *Per Capita* family income (RMB)			−7.06^c^	<0.001
< 3,000	115 (27.4%)	212 (47.6%)		
3,000–6,000	162 (38.6%)	155 (34.8%)		
> 6,000	143 (34.0%)	78 (17.5%)		
Chronic illness			−9.48^b^	<0.001
No	171 (40.7%)	127 (28.5%)		
Yes	249 (59.3%)	318 (71.5%)		
Family doctor contract			−5.99^b^	<0.001
No	313 (74.5%)	247 (55.5%)		
Yes	107 (25.5%)	198 (44.5%)		

a = Chi-square test; b = Independent samples *t*-test; c = Wilcoxon rank-sum test.

### 3.2 Analysis of positive and negative influencing factors for choosing community healthcare

**Figure 1:**
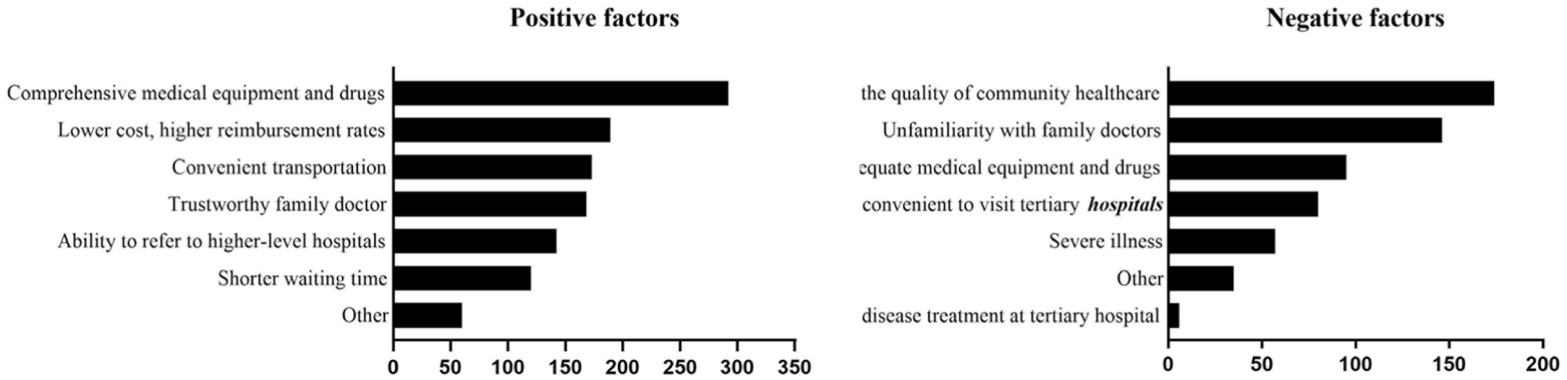
Positive and negative factors affecting community medical care.

An analysis of subjective factors influencing patients’ choice of community health service centers as their first point of contact revealed the following main positive factors (selection rate > 40%): comprehensive availability of diagnostic equipment and medications (46.9%), lower treatment costs and higher reimbursement rates (45.0%), and convenient transportation (41.2%). The primary negative factors (selection rate > 30%) were: lack of trust in the medical competence of the community health centers (39.1%) and unfamiliarity with their family doctor (32.8%) (Figure 1).

### 3.3 Comparison of awareness of the family doctor system between signed patients in both groups

A survey on the awareness of the family doctor system among patients who had signed up for family doctor services revealed that community patients scored significantly higher than tertiary hospital patients across four dimensions: acceptance of promotional efforts, understanding of the content of the agreement, awareness of the benefits of the signed services, and willingness to use the signed services (Table 2).

**Table 2:** Comparison of awareness of the family doctor system between tertiary hospital patients and community health center patients (^−^X ± S).

Items	Tertiary hospital patients’ scores (*n* = 107)	Community health center patients’ scores (*n* = 198)	*Z*-value	*p*-value
Acceptance of publicity methods	2.90 ± 1.16	4.27 ± 0.88	16.32	<0.001
Understanding of contract content	3.02 ± 1.16	3.78 ± 1.10	9.8	<0.001
Perception of contract service benefits	4.08 ± 0.94	4.25 ± 0.84	2.32	0.04
Willingness to use contract services	3.52 ± 0.95	4.48 ± 0.43	15.51	<0.001

### 3.4 Analysis of factors influencing primary care visits among tertiary hospital patients

Of the 420 patients treated at the tertiary hospital, 106 (25.2%) had initially sought treatment at a primary healthcare institution for their current illness. A binary logistic regression analysis was conducted, using whether the patient first sought treatment at a primary healthcare institution for the current illness as the dependent variable (coded as: Yes = 0, No = 1), and age, educational level, average monthly household income per capita, family doctor enrolment, and awareness of the tiered diagnosis and treatment system as independent variables. The results showed that age (>60 years) and awareness of the tiered diagnosis and treatment system were significant factors influencing whether tertiary hospital patients first sought care at a primary healthcare institution for their current illness (*p* < 0.05) (Table 3).

**Table 3:** Logistic regression analysis of factors influencing tertiary hospital patients’ choice of primary care institutions.

Independent variables	*B*	SE	Wald *χ*^2^	*p*-value	OR (95% CI)
Age (years, reference: 18–44)					
45–59	0.4	0.298	1.799	0.18	1.492 (0.831, 2.678)
≥60	0.664	0.328	4.101	0.043	1.942 (1.022, 3.690)
Education level (reference: primary school or below)					
Junior high school	0.502	0.614	0.669	0.413	1.652 (0.496, 5.501)
High school, technical school	0.141	0.566	0.062	0.804	1.151 (0.380, 3.489)
Undergraduate or above	0.272	0.547	0.248	0.619	1.313 (0.449, 3.838)
Per capita monthly household income (RMBreference: <3,000)					
3,000–6,000	0.062	0.303	0.041	0.839	1.064 (0.587, 1.927)
>6,000	−0.211	0.313	0.454	0.5	0.810 (0.439, 1.495)
Contracted family doctor (reference: contracted)					
Not contracted	0.283	0.26	1.184	0.277	1.327 (0.797, 2.207)
Awareness of hierarchical diagnosis system (reference: unaware)					
Aware	0.91	0.238	14.661	0	2.484 (1.559, 3.958)

## 4 Discussion

This study compared the healthcare-seeking behavior of patients at a tertiary hospital and a community health service center in Chengdu, analyzing the factors influencing their choices. Patients with higher education levels and household incomes were more likely to seek treatment at tertiary hospitals, consistent with previous studies (25–27). This indicates that socioeconomic status significantly affects patients’ preference for higher-level medical resources. In contrast, community health service center patients had higher rates of family doctor enrolment and chronic disease management, underscoring the growing role of community centers in managing chronic diseases. However, the family doctor system requires further promotion and enhancement.

### 4.1 Positive and negative factors affecting patient choices

The availability of comprehensive equipment, lower costs with higher reimbursement, and convenient transportation emerged as key positive factors for choosing healthcare institutions (28, 29). Conversely, the primary negative factor was a lack of trust in the medical competence of community institutions. This finding highlight that improving the quality of care and strengthening cost control mechanisms are pivotal for increasing the utilization of primary healthcare services (30). Policymakers should prioritize these aspects to advance the tiered healthcare system.

### 4.2 Family doctor system awareness and enrolment

While community patients demonstrated higher awareness and enrolment rates in the family doctor system compared to tertiary hospital patients, the overall enrolment remained low (44.5 and 25.5%, respectively). Additionally, some patients were unaware of their enrolment. Notably, family doctor enrolment was not a significant factor in patients’ decision to use primary care as the first point of contact (OR = 1.327; CI: 0.797, 2.207). Instead, awareness of the tiered healthcare system played a more critical role (OR = 2.484; CI: 1.559, 3.958).

### 4.3 Regional and international comparisons

The implementation of tiered healthcare varies widely across regions in China. Coastal areas like Shandong and Guangdong, with better-developed healthcare infrastructure, have achieved higher success (31). In contrast, less developed western and central regions face challenges due to insufficient resources and urban–rural disparities, with the most pronounced gap observed in the western region (56.70%) compared to the eastern region (26.04%) (31). This imbalance exacerbates patient concerns about primary healthcare quality and reduces the effectiveness of tiered care (32). Similar challenges exist globally, but countries like the UK, Denmark, and Australia demonstrate how robust family doctor systems can alleviate such issues. For instance, over 90% of UK residents annually consult family doctors who act as health gatekeepers (33–35). This contrasts sharply with China, where cultural factors and patient preferences for direct specialist access hinder the system’s development (36–38).

### 4.4 Challenges and recommendations for the family doctor system

China’s family doctor system faces challenges including low public awareness, inconsistent service quality, and limited collaboration between family doctors and specialists (39). These issues erode patient trust in primary care. Drawing on international examples, several strategies can be proposed: (1) Enhancing Awareness: Nationwide campaigns, like the UK’s NHS initiatives, could educate the public about the benefits of family doctors. An integrated electronic health record system could further build trust by demonstrating the continuity of care (33). (2) Strengthening Capabilities: Denmark’s professional training for family doctors and its collaborative referral networks offer a model for improving the quality and trustworthiness of primary care providers (34). (3) Optimizing Incentives: Capitation payment models in the UK and Australia incentivize family doctor enrolment and engagement (40, 41). Tailoring similar financial policies to China’s context could encourage greater participation.

### 4.5 Future directions

Future reforms should address resource disparities, particularly in rural and western regions. Policies should improve primary care accessibility, enhance general practitioner training, and introduce differentiated health insurance reimbursement structures to prioritize primary care visits. Additionally, further research should explore barriers to family doctor services and refine strategies to increase patient trust and system utilization (42–48).

## 5 Conclusion

This study reveals significant factors influencing patients’ healthcare-seeking behaviors in Chengdu. Patients with higher education levels and incomes prefer tertiary hospitals, while community health service centers play an essential role in managing chronic diseases and promoting family doctor enrolment. However, low trust in community healthcare and limited awareness of the family doctor system remain key barriers to the effective implementation of tiered healthcare.

To strengthen the tiered healthcare system, it is crucial to enhance the quality and accessibility of primary care services, address regional disparities in medical resources, and improve public awareness and trust in family doctors. Drawing on international experiences, such as those in the UK and Denmark, could provide valuable strategies for refining the family doctor system and promoting primary care utilization.

Future efforts should focus on integrating health insurance reforms, optimizing resource allocation, and enhancing general practitioner training. These measures are critical for advancing the equity and efficiency of China’s healthcare system, ensuring the sustainable implementation of tiered care.

### 5.1 Limitation

This study has several limitations. First, participants were sourced from a limited number of medical institutions in Chengdu, which may affect the generalisability of the findings. Expanding the sample to include diverse regions and institutions is necessary for validation. Second, reliance on self-reported questionnaire data may introduce response bias due to varying educational levels and comprehension among patients. Combining quantitative and qualitative methods could provide more robust insights. Lastly, the study did not fully explore the underlying barriers to patients’ awareness and utilization of the family doctor system, nor did it address systemic challenges in inter-institutional collaboration. Future research should investigate these aspects to support the optimization of tiered healthcare delivery.

## Data Availability

The original contributions presented in the study are included in the article/supplementary material, further inquiries can be directed to the corresponding author.
